# Daily physiological goal-setting: medical prescription and nursing adherence in a London teaching hospital ICU

**DOI:** 10.1186/cc9898

**Published:** 2011-03-11

**Authors:** MK Khan, S Alawad, M Thavasothy

**Affiliations:** 1 Royal London Hospital, London, UK

## Introduction

Individual, clearly defined physiological goal-setting can help to optimize patient care [1]. At our ICU, eight physiological goals can be prescribed on the daily ICU observation chart. These include Hb, MAP, ICP, CPP, pO_2_, pCO_2_, fluid balance and sedation scores. We performed a prospective audit assessing doctors' compliance with goal-setting, nursing adherence to these, and what goals nurses used when none were documented.

## Methods

An audit was carried out from December 2009 to March 2010. A total of 90 bedside charts were reviewed at random. Data collected included the total number of goals specified by the ICU medical team, the percentage of time those goals were achieved and what goals nurses set themselves if no goal had been previously documented.

## Results

Goals were prescribed for only 53% of patients. Most commonly prescribed were CPP targets for 63% of those with ICP bolts. The remaining parameters were prescribed for between 17 and 38% of patients, with balance and sedation goals being least commonly specified. For the ARDS subgroup of patients, no fluid balance goals were documented. Certain patterns were also evident; for example, pCO_2 _goal was more commonly stipulated for patients in the neuro group (for 41% of the group). However, there was no pattern seen in the number of goals specified per patient or according to the length of patient stay on the ICU. When goals were set, all targets were met 62% of the time, with >80% of targets met 79% of the time. When goals were not documented, however, 46% (pCO_2_) to 78% (fluid balance) of nursing staff were unable to specify what range of parameters they aimed to keep within. The remainder that did aim for particular target ranges stated they were aiming for physiologically normal parameters. Whilst sensible, this may not have been appropriate for some patients. For example, for fluid balance, a small number aimed for goals specified on previous days but 78% did not aim to achieve any goal, occasionally resulting in an inappropriately positive fluid balance.

## Conclusions

Adherence with physiological goal prescription amongst doctors is poor. When goals have been set, nursing adherence to them is very good. However, when no goals have been set, the physiological parameters that nursing staff aim for can be both variable and inappropriate, potentially resulting in both increased morbidity and prolonged length of stay on the ITU.
